# Assembly of DNA Architectures in a Non-Aqueous Solution

**DOI:** 10.3390/nano2030275

**Published:** 2012-08-31

**Authors:** Amethist S. Finch, Christopher M. Anton, Christina M. Jacob, Thomas J. Proctor, Dimitra N. Stratis-Cullum

**Affiliations:** 1 RDRL-SEE-B, Adelphi, MD 20783, USA; Email: christina.m.jacob@us.army.mil (C.M.J.); thomas.j.proctor7.ctr@mail.mil (T.J.P.); dimitra.n.stratis-cullum.civ@mail.mil (D.N.S.-C.); 2 Episensors, Inc., 590 Territorial Drive, Bolingbrook, IL 60440, USA; Email: canton@episensors.us

**Keywords:** DNA nanostructures, bioelectronics, biodirected assembly, CTAC, DNA

## Abstract

In the present work, the procedures for the creation of self-assembled DNA nanostructures in aqueous and non-aqueous media are described. DNA-Surfactant complex formation renders the DNA soluble in organic solvents offering an exciting way to bridge the transition of DNA origami materials electronics applications. The DNA retains its structural features, and these unique geometries provide an interesting candidate for future electronics and nanofabrication applications with potential for new properties. The DNA architectures were first assembled under aqueous conditions, and then characterized in solution (using circular dichroism (CD) spectroscopy) and on the surface (using atomic force microscopy (AFM)). Following aqueous assembly, the DNA nanostructures were transitioned to a non-aqueous environment, where butanol was chosen for optical compatibility and thermal properties. The retention of DNA hierarchical structure and thermal stability in non-aqueous conditions were confirmed via CD spectroscopy. The formation and characterization of these higher order DNA-surfactant complexes is described in this paper.

## 1. Introduction

Rapid development in electronic materials manufacturing has led to the development of standard devices with sub-100 nm features. These advances in science and engineering are remarkable but have reached a fundamental limit using a top-down approach, leading researchers to look towards nature to realize the next level of technological innovation [1,2]. Structural DNA nanotechnology has seen the development of a rich toolbox of building blocks over the last two decades [3,4]. These tools allow for the creation of a variety of structural architectures with distinct mechanical properties, and allow the assembly of a variety of 2D and 3D structures with exquisite control of periodicity and complexity [5,6,7,8,9,10,11].

Specifically, DNA has been a focus of many studies because it is well-characterized, relatively inexpensive, easy to synthesize, self-sorting, and readily modified with a variety of functional groups. Additionally, DNA is programmable at the molecular level with the ability to pre-organize into well-defined secondary and tertiary structures, and its size is to scale with traditional nanomaterial building blocks [2]. DNA assemblies range from simple geometric shapes to more complex higher order structures, all varied by use of the genetic code. These characteristics make DNA an ideal target for use as templates for materials applications, and for biologically enabled electronics and devices such as capacitors [12], sensors [13], wires [14,15,16], electro-optic modulators [17,18], waveguides [19], semi-conductor materials [20], electromagnetic interference (EMI) shielding materials [21], field effect transistors (FET) [22], energy storage materials [23], and organic light emitting diodes (OLED) [24,25].

Despite the promise of DNA assembly, a major hurdle in the use of assembled DNA architectures lies in fact that DNA architectures rely on aqueous phase assembly and are, thereby, not amenable to traditional manufacturing processes [26]. Development of methodologies that enable non-aqueous assembly of biological materials would address this key barrier to successful coupling of biological materials and their use in manufacturing of electronic devices. In addition, the ability to transition these DNA architectures into organic solvents should allow these materials to be used as interesting alternatives to traditional dielectrics since the interface and surface morphology seems to play a crucial role in device performance [27]. In this paper, we will address a critical issue of the biological interface by complexing the DNA with a surfactant, which allows it to be soluble in a variety of non-aqueous solvents and greatly increases its thermal stability. This is the first report of higher order DNA nanoarchitectures assembled in non-aqueous conditions in literature; these procedures are necessary in avoiding unwanted oxidation of certain electronic substrates and for providing added mechanical stability to the biological materials.

## 2. Results and Discussion

For these experiments, a DNA-cationic surfactant complex was created using either hexadecyltrimethylammonium-chloride (CTAC) or bromide (CTAB). This DNA-surfactant complex (DNA-CTMA) rendered the DNA water insoluble, causing it to precipitate out of its aqueous solution, and also had the added benefit of making the DNA more mechanically and thermally stable [22]. The DNA-CTMA complex was soluble in a variety of non-aqueous solvents, including isopropanol, ethanol, methanol, acetonitrile, and butanol [28]. Previous CD studies have shown that when using genomic DNA, the DNA-CTMA compound retains its double-helical structure in these solvents at temperatures over 100 °C [19].

Many different research laboratories have published sequences and protocols for creating a large variety of shapes and structures using differing numbers of DNA strands and different strand lengths [3]. For this work, the wagon wheel DNA (wwDNA) was selected as the initial template, primarily due to its overall size [29]. Specifically, wagon wheel templates have a void size of 10 nm or less and have a relatively large feature size (>15 nm), facilitating visualization of the samples by AFM. Furthermore, this template only consists of two DNA strands that combine to form a higher order structure using a T-junction motif, which greatly simplifies the protocol for creating the initial template. To demonstrate the versatility of this approach, several other T-junction motif-based DNA architectures were used, illustrating compatibility with structural diversity and ease of assembly.

**Figure 1:**
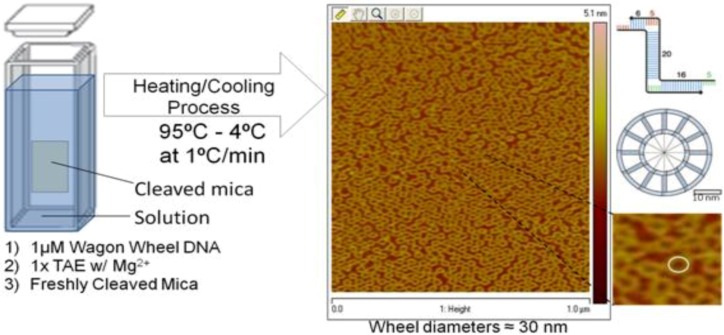
Schematic of the preparation of the wagon wheel DNA (wwDNA) in a cuvette for simultaneous monitoring of circular dichroism (CD) and UV spectra, as well as further characterization via atomic force microscopy (AFM).

In order to have a baseline comparison to a non-traditional solvent system, the wwDNA construct was first fabricated in aqueous solution, and characterized using AFM and CD spectroscopy (Figure 1). The wwDNA samples were co-incubated in aqueous solution with freshly cleaved mica and then imaged via AFM. While, the vast majority of absorbing species are still in solution, this co-incubation procedure allows for direct surface AFM characterization of the samples visualized by CD in solution.

**Figure 2:**
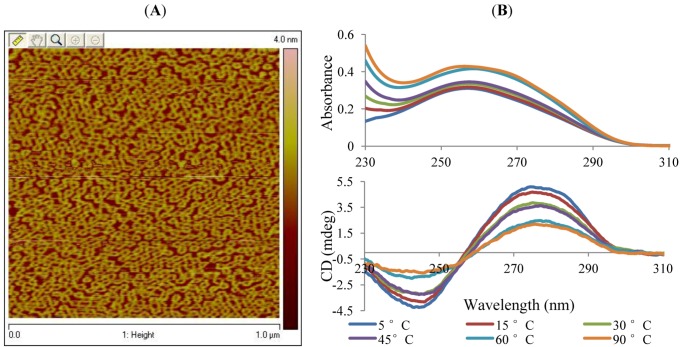
wwDNA formation monitored via surface (2A) and solution (2B) techniques (**A**) AFM of sample prepared by surface mediated assembly of wwDNA architecture on freshly cleaved mica in aqueous solution (TAE-Mg^2+^); (**B**) CD and UV spectroscopy of wwDNA in aqueous solution with respect to increasing sample temperature; where TAE is Tris-Acetate-EDTA.

As illustrated in Figure 2A, the size of the features are in good comparison with literature reports and are approximately 30 nm in diameter, with a 10 nm void, and a 2.5 nm feature height [29]. In addition, upon further AFM scanning, it appears that >90% of the sample was covered in a similar packing density to that illustrated in Figure 2A, which is also consistent with the reported literature of template-assisted DNA self-assembly on mica [29,30]. These initial results led to further experiments to allow for monitoring of the wwDNA assembly process in solution.

Although the AFM allowed for verification of assembly on the mica surface, the technique does not allow for studying the process in solution. We determined that AFM and CD spectroscopy data could be coupled and used as complementary techniques in order to measure the assembly simultaneously in solution. The wwDNA samples were co-incubated in aqueous solution with freshly cleaved mica, and this mica-DNA solution was monitored via CD and UV spectroscopy and then the mica was imaged via AFM. Figure 2B illustrates our ability to monitor the assembly of higher order wwDNA structures in solution via CD with simultaneous monitoring of DNA concentration via UV spectroscopy. This simultaneous monitoring illustrates that changes in the CD spectra are due to conformation changes, and are independent of sample concentration. As the temperature of the sample decreased slowly over time, the secondary structural features of the DNA became more prevalent, as evidenced by the characteristic alpha helical structure on the CD [31]. Following development of the CD methodology for solution assembly studies and characterization of the formation of the wwDNA structures in an aqueous environment, the methods were used to enable assembly of the wwDNA-CTMA complex under non-aqueous conditions.

In order to directly compare results with the published literature, experimental techniques were first validated using a more widely studied DNA system, genomic salmon sperm DNA (saDNA) [19]. The saDNA samples were reacted with two different surfactant solutions (CTAB/CTAC) at different concentrations, in order to determine the optimum conditions for experiments. These saDNA experiments illustrated that the CTAC was more appropriate due to its water solubility at room temperature. Additionally, 1-Butanol was chosen as the non-aqueous solvent for all experiments due to its high boiling point and lack of absorbance in the active CD spectroscopy region. Once the DNA-surfactant complex formed, the saDNA-CTMA samples were characterized via CD spectroscopy and AFM, with 1-butanol as the solvent (Figure S1 in supporting information). The CD confirmed literature reports andillustrates that the structure of the saDNA does not change upon creation of the saDNA-CTMA complex, as shown by the characteristic dsDNA CD spectra [31,32]. These results and experimental controls conducted with the saDNA provided a standard sample for comparison with the wwDNA.

**Figure 3:**
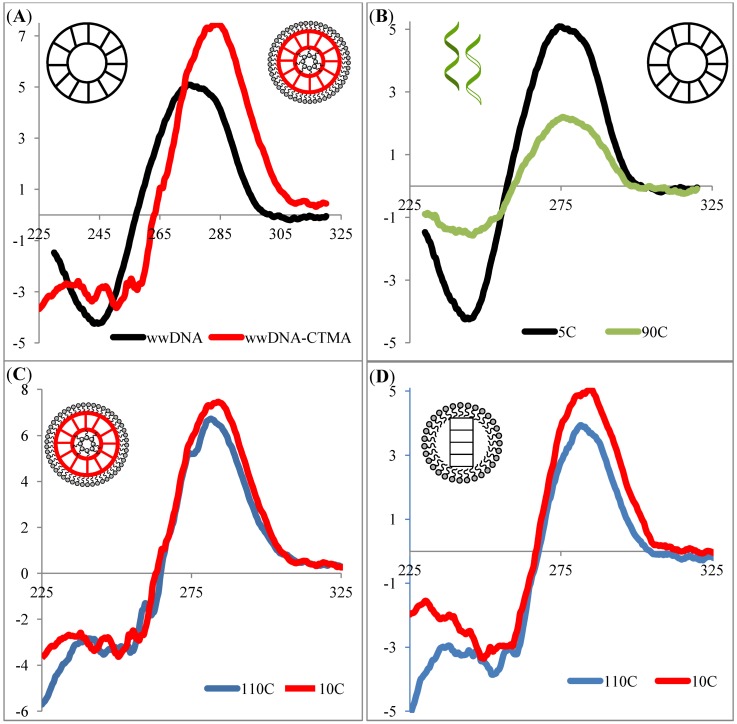
CD Spectroscopy (**A**) wwDNA sample in aqueous solution (black) *vs.* wwDNA-hexadecyltrimethylammonium (wwDNA-CTMA) complex in butanol (red) at 10 °C; (**B**) wwDNA in aqueous solution at 5 °C (black) *vs.* 90 °C (green); (**C**) wwDNA-CTMA in butanol at 10 °C (red) *vs.* 110 °C (blue); (**D**) Ladder DNA-CTMA in butanol at 10 °C (red) *vs.* 110 °C (blue).

The experimental procedures outlined for the saDNA-CTMA characterization were repeated with the wwDNA-CTMA. As illustrated in Figure 3A, the CD spectrum of wwDNA-CTMA in butanol suggests that the double helical structure of DNA was preserved in the wwDNA-CTMA complex, as illustrated by the lack of change in the shape of the CD spectra.

Although the spectrum from aqueous to butanol is slightly shifted, this shift is in agreement with literature reports that the overall shape of the spectra remains the same, suggesting retention of overall structure [31,32]. For comparison, Figure 3B illustrates the differences between the high and low temperature CD spectra of the wwDNA in aqueous solution. The wwDNA-CTMA in butanol was then subject to the same thermal melting conditions as the aqueous wwDNA sample. As illustrated in Figure 3C, no loss of features or loss of helical structure is detected with increased temperature up to the limits of the instrument (110 °C). This thermal melting experiment was repeated with the ladder DNA-CTMA sequence in butanol (Figure 3D), illustrating the ubiquity of this technique for transitioning DNA architectures to non-aqueous solvents.

**Figure 4:**
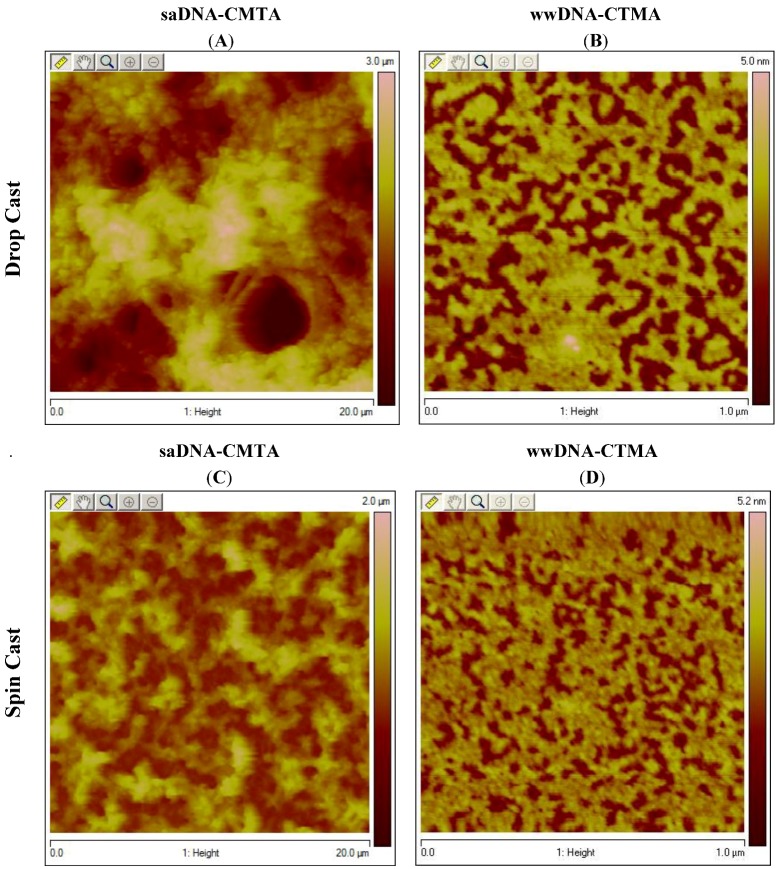
Salmon sperm DNA-CTMA (saDNA-CTMA) and wwDNA-CTMA complex were either drop cast or spin onto freshly cleaved mica and imaged via AFM. (**A**) saDNA-CTMA dropcast; (**B**) wwDNA-CTMA drop cast; (**C**) saDNA-CTMA spin cast; (**D**) wwDNA-CTMA spin cast.

In addition to the CD spectroscopy, AFM of the saDNA-CTMA and wwDNA-CTMA was conducted to further confirm sample morphology upon reconstitution with surfactant. Figure 4 illustrates the differences in the genomic saDNA samples (Figure 4A,C) versus the programmed architecture wwDNA samples (Figure 4B,D). It is evident by the size of the features in Figure 4A,C that in the genomic saDNA-CTMA sample appears to form much larger micron-sized aggregates in butanol. Whereas, the wwDNA-CTMA sample illustrated in Figure 4B,D appears to retain its much smaller nanometer sized structure in butanol. The DNA samples were both spin cast and drop cast onto freshly cleaved mica in order to illustrate that the differences in the relative feature size between the samples was not due to any differences in sample processing.

These preliminary results are exciting and suggest that, although further optimization is necessary, these techniques should allow for direct utilization of structurally programmable DNA as materials and templates in electronics manufacturing processes.

## 3. Experimental Section

### 3.1. Materials

RP-HPLC purified oligonucleotides were purchased from IDT DNA (Coralville, IA, USA). DNA samples were reconstituted to a 100 μM final concentration using 1X IDTE (IDT DNA). All aqueous solutions were prepared with nanopure water (ddH_2_O). All chemicals and supplies were purchased from Sigma-Aldrich or Invitrogen, and were of the highest grade and purity available. DNA sequences were chosen for their secondary structural characteristics as described in the literature [29].

### 3.2. Methods

*DNA Wagon Wheel Template Reconstitution*: The specific DNA sequence was synthesized by IDT and packaged separately as single-stranded DNA, dubbed “Wheel 1” (sequence: 5'-TCC ACG GTC TGC TAC TCG GTA ATG GCT CAT CAA GCG TCC AGT TCC GCA AAC G-3') and “Wheel 1 comp” (sequence: 5'-CGC TTC GTT TGC GGA ACT GGA GAT GAG CCA TTA CCG AGT AGG TGG ACA GAC C-3').

The reconstitution of the two single strands of DNA into the double-stranded, tertiary wheel structure was accomplished with the use of a JASCO J-815 CD spectrometer (Easton, MD, USA). 1X Tris-Acetate-EDTA (TAE) was diluted from 10X stock solution and combined with 12.5 mM Mg^2+^ (1 M stock), 2.5 µL Wheel 1 (100 µM stock in TAE), and 2.5 µL Wheel 1 Comp (100 µM stock in TAE), and samples (with and without freshly cleaved mica present) were incubated in a cuvette for CD studies and a microfuge tube for all other experiments. The wagon wheel complex, once formed, is referred to as wwDNA. As seen in Figure 1, the mica disk was placed in a cuvette during its reconstitution process so as to provide even coating over the entire mica surface. The sample was heated to 95 °C and then cooled to 4 °C at a ramp rate of 0.1 °C per minute.

*Creating the DNA-Surfactant Complex *(*DNA-CTMA*): The process of the DNA-CTMA reaction has been previously studied and explained, and is only being reproduced in this experiment [32]. Both CTAB and CTAC were reacted with the reconstituted DNA (salmon sperm and wagon wheel DNA) to form a complex that appeared as a white, solid pellet that was found to be insoluble in water. The samples were then centrifuged for 5 min at a speed of 7500 rpm and at a temperature of 4 °C. The resulting supernatant was placed in a collection tube to determine efficiency of complex formation, and the pellet was rinsed with ddH_2_O. The centrifugation, supernatant collection, and rinsing steps were repeated two additional times. The samples were placed in an Eppendorf Vacufuge (Hauppauge, NY, USA) and lyophilized for about 1 h until samples were dry. The CTAC was chosen over CTAB for future experiments because it is completely soluble at room temperature, whereas the CTAB precipitates at room temperature in aqueous solution and requires an additional heating step.

*CD Spectroscopy*: The formation of the wagon wheel template in both aqueous and non-aqueous solutions was monitored via CD spectroscopy using a Jasco 850 CD spectrometer (Easton, MD, USA), using the temperature interval program. The temperature interval program takes a CD and UV scan at preset temperature intervals (as determined by the user), allowing for direct monitoring of the sample at different temperatures. In addition to direct monitoring of the DNA and DNA-CTMA complex formation, the supernatant of each sample in the DNA-CTMA preparation was analyzed using both CD and UV spectrometry in order to determine the efficiency of the DNA-CTMA complex formation. The absorbance for each sample was tracked to determine the remaining concentrations of DNA left in the supernatant after rinsing the pellet with water.

*Testing Solubility in Butanol*: Lyophilized sample pellets were resuspended in different organic solvents: Isopropyl alcohol, ethanol, methanol, acetonitrile, and butanol [28]. Butanol was chosen as the primary solvent for experiments due to its high boiling point and optical properties.

*Sample Mounting*: The samples were mounted on silicon wafers or incubated with freshly cleaved mica in solution. The DNA and DNA-CTMA samples on the silicon wafers were either spin-cast or drop-cast onto the silicon surface. To spin-cast the sample, about 150 µL of the DNA sample was placed onto a silicon wafer and spun in a Laurell Technologies Corporation Spin Processor (North Wales, PA, USA) at speeds ranging from 1000 to 8000 rpm. For drop-casting, 20–50 µL of the DNA sample was added drop-wise by micropipette onto a silicon wafer and the liquid was allowed to either evaporate under room temperature conditions or samples were incubated for 30 minutes followed by a rinse with ddH_2_O and dried under N_2_. Sample uniformity and surface roughness was then characterized by AFM and SEM.

*Surface Characterization of DNA-Surfactant Complex by Atomic Force Microscopy *(*AFM*): Veeco Nanoman V SPM Atomic Force Microscope (Santa Barbara, CA, USA), was used in tapping mode with Bruker OTESPA tips (Santa Barbara, CA, USA) to image the DNA samples.

## 4. Conclusions

To summarize, these results show for the first time, formation of higher order DNA structures, both in aqueous and non-aqueous solutions. Characterization was performed via AFM (surface) and CD spectroscopy (solution). The aqueous wwDNA samples were combined with surfactant CTAC to form DNA-CTMA complexes, which were soluble in a variety of organic solvents. Future work will include optimization of the material surface for single layer deposition of the templated DNA-CTMA complex, as well as transitioning to a wide range of structures. These nanostructured materials have great promise in creating bottom-up features smaller than those created by current top-down lithographic techniques.

## Supplementary Materials

Supplementary File 1
