# Supplementary material for: Assembly of DNA Architectures in a Non-Aqueous Solution
Source: Nanomaterials (Basel). 2012 Aug 31;2(3):275–85. doi: 10.3390/nano2030275 (PMC5304584; doi:10.3390/nano2030275)

Supporting Information

Assembly of DNA Architectures in a Non-Aqueous Solution

Amethist S. Finch [1,*], Christopher M. Anton [2], Christina M. Jacob [1], Thomas J. Proctor [1] and Dimitra N. Stratis-Cullum [1]

[1] RDRL-SEE-B, Adelphi, MD 20783, USA; E-Mails: christina.m.jacob@us.army.mil (C.M.J.); thomas.j.proctor7.ctr@mail.mil (T.J.P.); dimitra.n.stratis-cullum.civ@mail.mil (D.N.S.C.)

[2] Episensors, Inc., 590 Territorial Drive, Bolingbrook, IL 60440, USA; E-Mail: canton@episensors.us

* Author to whom correspondence should be addressed; E-Mail: amethist.s.finch.civ@mail.mil; Tel.: +1-301-394-0326.

Figure S1. CD spectroscopy of SaDNA (red) in aqueous solution and saDNA-CTMA (blue) in butanol.

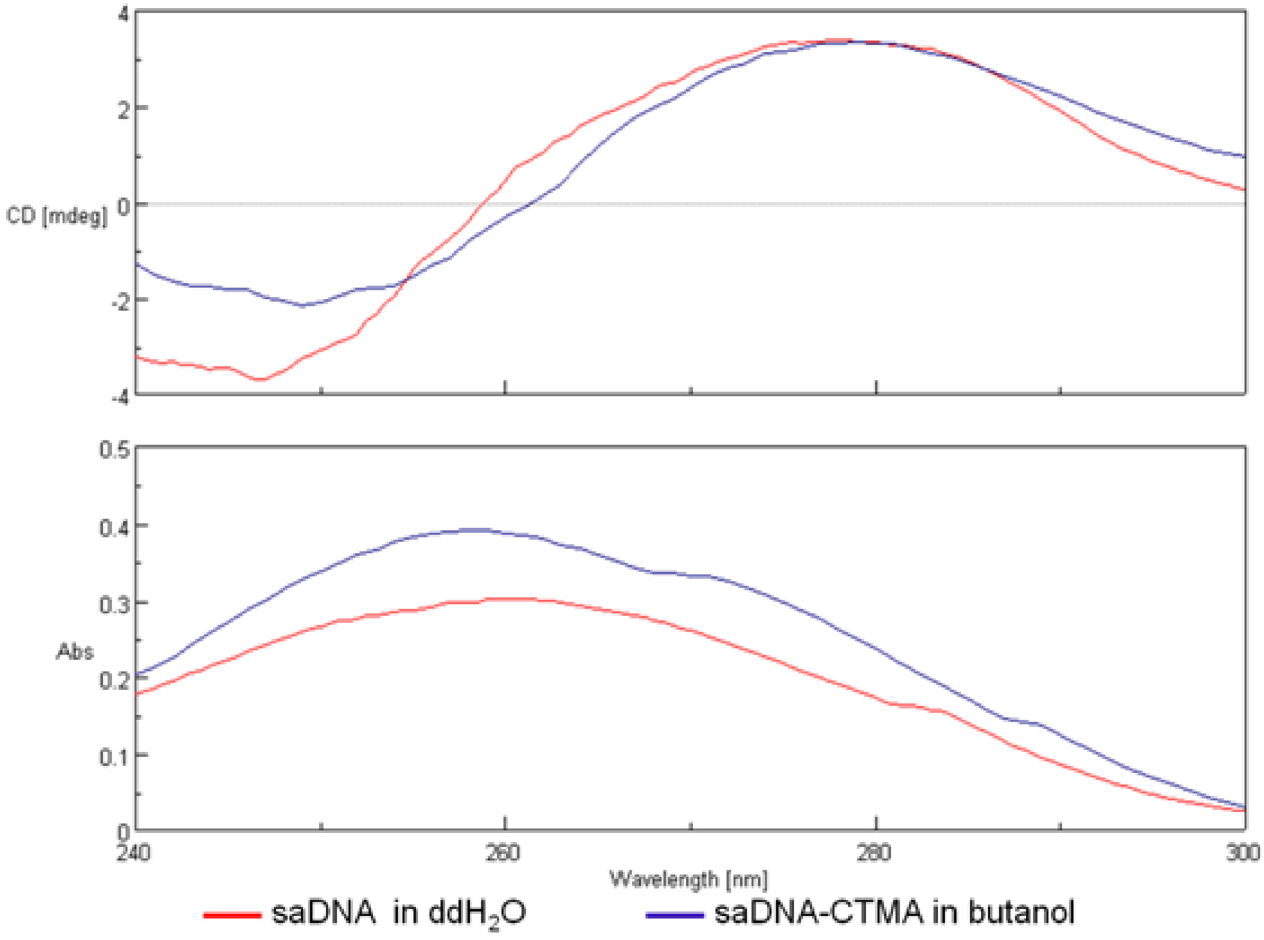

Supplement: Supplementary File 1 [file nanomaterials-02-00275-s001.pdf]
